# Cortical Implication in Lower Voluntary Muscle Force Production in Non-Hypoxemic COPD Patients

**DOI:** 10.1371/journal.pone.0100961

**Published:** 2014-06-27

**Authors:** Francois Alexandre, Nelly Heraud, Nicolas Oliver, Alain Varray

**Affiliations:** 1 Movement To Health Laboratory, Euromov, University of Montpellier 1, Montpellier, France; 2 Clinique du Souffle La Vallonie, Fontalvie, Lodève, France; University of Rome Foro Italico, Italy

## Abstract

Recent studies have shown that muscle alterations cannot totally explain peripheral muscle weakness in COPD. Cerebral abnormalities in COPD are well documented but have never been implicated in muscle torque production. The purpose of this study was to assess the neural correlates of quadriceps torque control in COPD patients. Fifteen patients (FEV_1_ 54.1±3.6% predicted) and 15 age- and sex-matched healthy controls performed maximal (MVCs) and submaximal (SVCs) voluntary contractions at 10, 30 and 50% of the maximal voluntary torque of the knee extensors. Neural activity was quantified with changes in functional near-infrared spectroscopy oxyhemoglobin (fNIRS-HbO) over the contralateral primary motor (M1), primary somatosensory (S1), premotor (PMC) and prefrontal (PFC) cortical areas. In parallel to the lower muscle torque, the COPD patients showed lower increase in HbO than healthy controls over the M1 (p<0.05), PMC (p<0.05) and PFC areas (p<0.01) during MVCs. In addition, they exhibited lower HbO changes over the M1 (p<0.01), S1 (p<0.05) and PMC (p<0.01) areas during SVCs at 50% of maximal torque and altered motor control characterized by higher torque fluctuations around the target. The results show that low muscle force production is found in a context of reduced motor cortex activity, which is consistent with central nervous system involvement in COPD muscle weakness.

## Introduction

Peripheral muscle dysfunction is very frequent in COPD and has major consequences. The loss of muscle force in COPD patients has become a matter of heightened concern because it implies exercise limitation [1], increased use of health care resources [2], and higher mortality [3]. The involvement of muscle atrophy in this loss was established several years ago [4]. However, several elements point to the existence of other explanatory mechanisms. For instance, a recent study reported that COPD patients exhibit a decline in muscle force even when their muscle mass is comparable to that of healthy controls [5]. In addition, the lower muscle force across GOLD stages (between GOLD I and IV) is not explained by smaller muscle cross-sectional areas [6]. Therefore, other mechanisms should be explored to enhance understanding of the pathophysiology of muscle weakness in COPD.

A decline in muscle force can be caused by alterations in the muscle and/or the nervous system [7]. Interestingly, several studies have assessed the cerebral properties in COPD patients and reported small cerebral vessel disease [8], gray matter deficits [9], white matter lesions [9], [10] and neuronal dysfunction [11]. At a more functional level, COPD patients exhibit lengthening peripheral [12] and central [13] nervous conduction times, alterations in motor cortex excitability [14], and cognitive disorders [9], [10]. In contrast, the potential repercussions over the central motor drive and muscle performance are unknown.

A few studies have evaluated muscle activation in COPD using the twitch interpolation technique [15]–[17], an indirect assessment of the central motor drive. However, the results were discrepant [15]–[17] and no definitive conclusions could be drawn. The discrepancies may be explained by the poor sensitivity of this technique at near maximal force, which makes it difficult to discriminate two populations during maximal voluntary contractions (MVCs) [18]. Thus, the question of nervous system involvement in COPD muscle weakness remains unanswered.

An alternative to circumvent the limitations of twitch interpolation could be the use of neuroimaging techniques. Force output is directly related to cortical activity as measured by functional magnetic resonance imaging (fMRI) [19] and functional near infrared spectroscopy (fNIRS) [20]. The fNIRS oxy- (HbO) and deoxy-hemoglobin (HbR) signals are strongly correlated with the blood-oxygen-level-dependent (BOLD) fMRI signal, and they are widely acknowledged to be reliable for functional cortical activity assessment in various conditions [21]–[23]. In addition, fNIRS has been validated for the study of neural activity in a wide range of populations, such as the elderly [23] and COPD [24], stroke [25], and obese patients [26] during various motor tasks, including MVCs [26]. Whereas fMRI restricts body movement within the enclosed chamber, fNIRS presents a high signal-to-noise ratio and relatively poor sensitivity to motion artifacts, making it the more suitable for cortical activity assessment during exercise [27], [28].

Given the numerous cerebral alterations in COPD that have never been linked with poor muscle force production, the purpose of this study was to assess the fNIRS-neural correlates of quadriceps contraction at maximal and submaximal intensity in COPD patients. We hypothesized lower activity over motor cortical areas in COPD patients than healthy controls during quadriceps contractions.

## Materials and Methods

### Subjects

Fifteen COPD patients and 15 age- and sex-matched sedentary healthy subjects were recruited for the study. The participation criteria for the COPD patients were forced expiratory volume in the 1^st^ second (FEV_1_) between 30 and 80% of the predicted values, with no exacerbation or weight loss in the month preceding the study. No patient had taken part in a rehabilitation program in the previous 12 months. The non-inclusion criteria for the participants were an inability to give written consent, inability to perform the experimental maneuvers, impaired visual function, use of drugs known to impair brain function, current or past alcohol abuse, and neurologic or neuromuscular disease. All participants gave written consent. Procedures were approved by the local Ethics Committee (Comité de protection des personnes Sud Est VI, number AU980) and complied with the principles of the Declaration of Helsinki for human experimentation. The study was registered at www.clinicaltrials.gov as NCT01679782.

### Design

**Figure 1:**
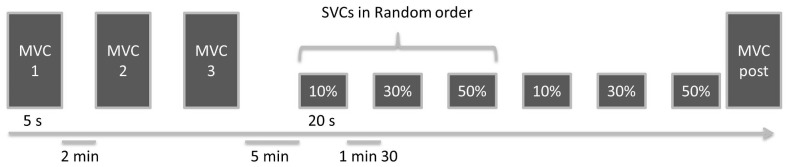
Experimental Design. MVC: Maximal Voluntary Contraction, SVC: Submaximal Voluntary Contraction.

All participants underwent a medical examination, including evaluation of resting pulmonary function, body composition and clinical parameters, before taking part in the study. The protocol consisted of maximal and submaximal voluntary contractions of the knee extensors, during which cortical activity was assessed non-invasively from changes in fNIRS signals [22], [25]. The exercise protocol is presented in Figure 1. After determination of the dominant leg, the participants performed a standardized warm-up of the knee extensors by repeating 20 submaximal voluntary contractions for 2 s every 5 s. They next performed three maximal voluntary contractions (MVCs) and three submaximal voluntary contractions (SVCs) at 10, 30 and 50% of the maximal voluntary torque twice in random order. Each MVC lasted for 5 s and two successive MVCs were separated by a 2-min resting period. Each SVC lasted for 20 s and two successive SVCs were separated by a 1.5 min resting period. The random draw to determine the order of the SVCs took place immediately after the three MVCs had been performed and the target torques calculated. A last MVC was performed to ensure the absence of neuromuscular fatigue at the end of the exercise testing.

### Mechanical recordings

Subjects were comfortably seated on a dedicated ergometer for knee extensor testing (Quadriergoforme, Aleo Industrie, Salome, France) with a 30° back inclination. Chair adjustments were made to ensure that the foot, patella and coxofemoral articulation of the dominant leg were in the same axis. The knee angle was set to 110°. The pelvis and the proximal extremity of the patella were securely attached to the chair in order to minimize movements of adjacent muscles. In addition, the head was supported by a neck brace to avoid potential head motion. Torque of the knee extensors during the contractions was recorded with a strain gauge torque sensor (Captels, Saint Mathieu de Treviers, France). The acquired analog signal was converted into digital data (DA conversion) through an acquisition system (Biopac MP100, Biopac Systems, Santa Barbara, CA, USA) and instantaneously relayed to a screen to give visual feedback. During each MVC and each SVC, subjects were verbally encouraged to ensure maximal muscle torque and to maintain the force requirement, respectively. Before the SVCs, the target torque was clearly indicated to the subjects via the computer monitor and they received visual feedback of their performance during the contractions.

### Cortical activity assessment

**Figure 2:**
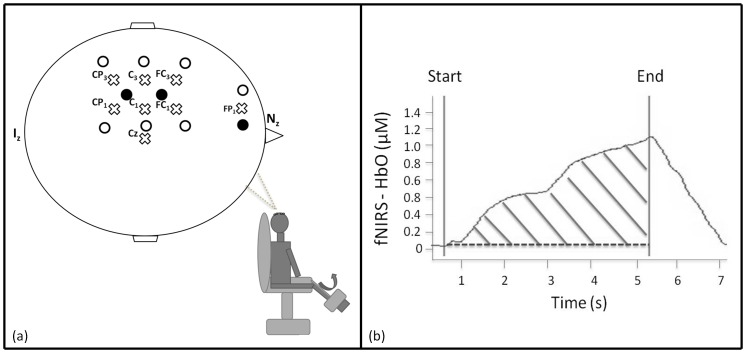
Measurement of cortical activity by functional near-infrared spectroscopy (fNIRS). a) fNIRS optode placement. Three receivers (black circles) and seven emitters (white circles) were set over the scalp, resulting in 9 measured channels. The crosses represent the reference points used to target primary sensory (CP_3_ - CP_1_), primary motor (C_3_ - C_1_), premotor (FC_1_ - FC_3_) and prefrontal cortical areas (FP_1_) according to the modified international EEG 10-10 system. I_z_: Inion, N_z_: Nasion.b) Example of a functional near-infrared spectrospcopy oxyhemoglobin signal (fNIRS-HbO) during a maximal voluntary contraction in one subject. Hatched area represents the area under the curve of HbO (as index of neural activity).

A continuous wave multichannel functional near-infrared spectroscopy (fNIRS) system (Oxymon Mark III, Artinis, the Netherlands) was used at two wavelengths in the near-infrared range (nominal wavelengths of 760 and 850 nm) to detect regional concentration changes in oxyhemoglobin (HbO) and deoxyhemoglobin (HbR) during cortical activation over cortical motor areas. fNIRS is based on neurovascular coupling: when neural activity increases, the increase in regional cerebral blood flow is ten times higher than the increase in regional oxygen consumption. Thus, as the increase in regional cerebral blood flow greatly exceeds the increase in oxygen consumption, neuronal hemodynamic concentration is closely coupled with the increase in regional cerebral blood flow, which turns into local hyperoxygenation [29] and subsequent increase in HbO with a decrease in HbR [30]. The fNIRS-measured hemoglobin is comparable to the BOLD-fMRI signal and mainly reflects changes in cortical gray matter hemodynamic [22]. The fNIRS optodes were held by a cap fixated by several bands surrounding the subject's head. A total of nine channels were positioned over the contralateral primary motor (M1), primary somatosensory (S1), premotor (PMC) and prefrontal (PFC) cortical areas in accordance with the modified EEG 10-10 system [31] (Figure 2a). The source-detector spacing was set to 3.5 cm. During probe placement, Oxysoft software (V6.0, Artinis, the Netherlands) allowed real time assessment of the quality of the fNIRS signal for each channel based on the light source power level and the receiver gain. Hemoglobin concentrations were corrected by implementing a specific differential pathlength factor (4.99+0.067× age^0.814^), in order to convert the concentration changes in HbO and HbR to M units [32]. The fNIRS signal was low-pass filtered (finite impulse response) using a cut-off frequency of 0.7 Hz. The sampling rate was set at 10 Hz. To avoid systemic bias, we also monitored the pulsed arterial oxygen saturation (SpO_2_) in a restricted group of patients (n = 12). The oximetry probe (Weinman, Hamburg, Deutshland) was placed on the index finger and the participants were asked to keep their hand motionless throughout the experiment.

### Data analysis

During MVCs, maximal quadriceps torque (Q_MVC_) was calculated over the highest 500-ms plateau of torque during the best trial of the three MVCs.

During SVCs, task matching was evaluated by averaging and comparing the mean performed torque versus the target torque. In addition, during each SVC, the motor control was assessed from the fluctuations around the target. An inaccuracy index (Inaccuracy_index_) was calculated and represents the RMS (root mean square) of the difference between produced and target torques during the 20 s of submaximal voluntary contractions expressed as a percentage of the target torque [33]. The normalization by the target torque is necessary because the torque variability is known to be proportional to the torque level [34].

Changes in cortical activity were determined from HbO variations as previously described [25]. HbO signals with artifacts or a too-low signal-to-noise ratio were marked and excluded from the analyses under a visual pre-processing analysis [35]. During the best trial of the three MVCs and of the more accurate SVCs at 10, 30 and 50% of Q_MVC_, the area under the curve of HbO normalized over time was used as an index of neural activity (Figure 2b).

The data, taken from the four channels over the M1 area, the two channels over the S1 area, and the two channels over the PMC area were averaged, resulting in the overall response of, respectively, the M1, S1 and PMC areas.

Before the beginning of exercise testing, resting HbO was calculated for each cortical area over a 2-min resting period, respecting the same analysis process as aforementioned.

### Statistical analysis

All statistical analyses were performed using Statistica software (StatSoft, Inc., version 6.0, Tulsa, OK, USA). All data were examined for normality using a Shapiro-Wilk test. Differences in subject characteristics and variables recorded during MVCs were tested between controls and patients using an unpaired Student's t-test. Absence of neuromuscular fatigue was tested using a two-way analysis of variance (ANOVA) with group as between-subject factor (COPD and controls) and condition (before and after exercise testing) as within-subject factor. The Inaccuracy_index_ and HbO recorded during SVCs were tested using a two-way ANOVA with group as between-subject factor and torque level (10, 30 and 50% of Q_MVC_) as within-subject factor. Analysis of covariance (ANCOVA) with adjustment for Q_MVC_ was used to ensure that the difference in HbO between patients and controls was not due to a difference in muscle torque between the groups. Task compliance during SVCs was tested with a three-way ANOVA with group as between-subject factor and condition (target versus performed) and torque level (10, 30 and 50% of Q_MVC_) as two within-subject factors. The underlying assumptions of ANOVA were checked using a Levene test (homogeneity of the variance) and a Mauchly test (sphericity of the variance). When the ANOVA F ratio was significant (p<0.05), the means were compared by a LSD post-hoc test. Data are reported as means and standard error of the mean (SE).

## Results

### Subject characteristics

The subject characteristics are given in Table 1. Consistent with the matching, no difference in the gender ratio or age was observed between patients and controls. Weight, body mass index and fat-free mass index exhibited no significant differences (p>0.05). According to the Voorrips questionnaire [36], the level of physical activity was comparable for patients and controls (p = 0.64).

**Table 1:** Characteristics of the subjects included in the study.

	Control(n = 15)	COPD(n = 15)	p-value
Gender M/F	10/5	10/5	
Age yrs	61 (2.9)	62.8 (2.5)	NS (0.64)
Weight kg	75.8 (3.3)	72.8 (4.2)	NS (0.57)
BMI kg.m^−2^	25.8 (1)	25.3 (1.3)	NS (0.76)
FEV_1_ L	3.1 (0.2)	1.5 (0.2)	<0.001
FEV_1_ % pred	104.5 (3)	54.1 (3.6)	<0.001
FEV_1_/FVC	73.1 (1.1)	49.7 (2.4)	<0.001
FFM kg	55.3 (3)	53.9 (3)	NS (0.73)
FFMI kg.m^−2^	18.6 (0.5)	18.8 (0.7)	NS (0.92)
Voorrips AU	7.4 (1.25)	6.5 (1.35)	NS (0.64)
PaO_2_ mmHg		72.9 (2.8)	
PaCO_2_ mmHg		37.4 (1.4)	

BMI: Body Mass Index, FEV_1_: Force Expiratory Volume in 1 s, FVC: Force Vital Capacity, FFM: Fat-Free Mass, FFMI: Fat-Free Mass Index. NS: no significant difference between controls and COPD patients. Values are mean (SE).

### Control of absence of desaturation and fatigue during exercise testing

SpO_2_ remained stable for all patients during both MVCs and SVCs. The mean ΔSpO2 was 0.01±0.12% during MVCs (p = 0.98) and 0.017±0.19% during SVCs (p = 0.98).

Absence of neuromuscular fatigue was checked by changes in Q_MVC_ after the protocol. Both patients and controls exhibited no significant differences in Q_MVC_ (condition and interaction *F* ratio ranged from 0.17 to 1.10, p ranged from 0.31 to 0.68).

### Maximal voluntary contractions

Q_MVC_ was significantly lower by 24.8% in COPD patients compared with controls (131.9±16.6 and 175.4±24.9 Nm, respectively, for patients and controls, t = 2.5, p<0.05).

**Figure 3:**
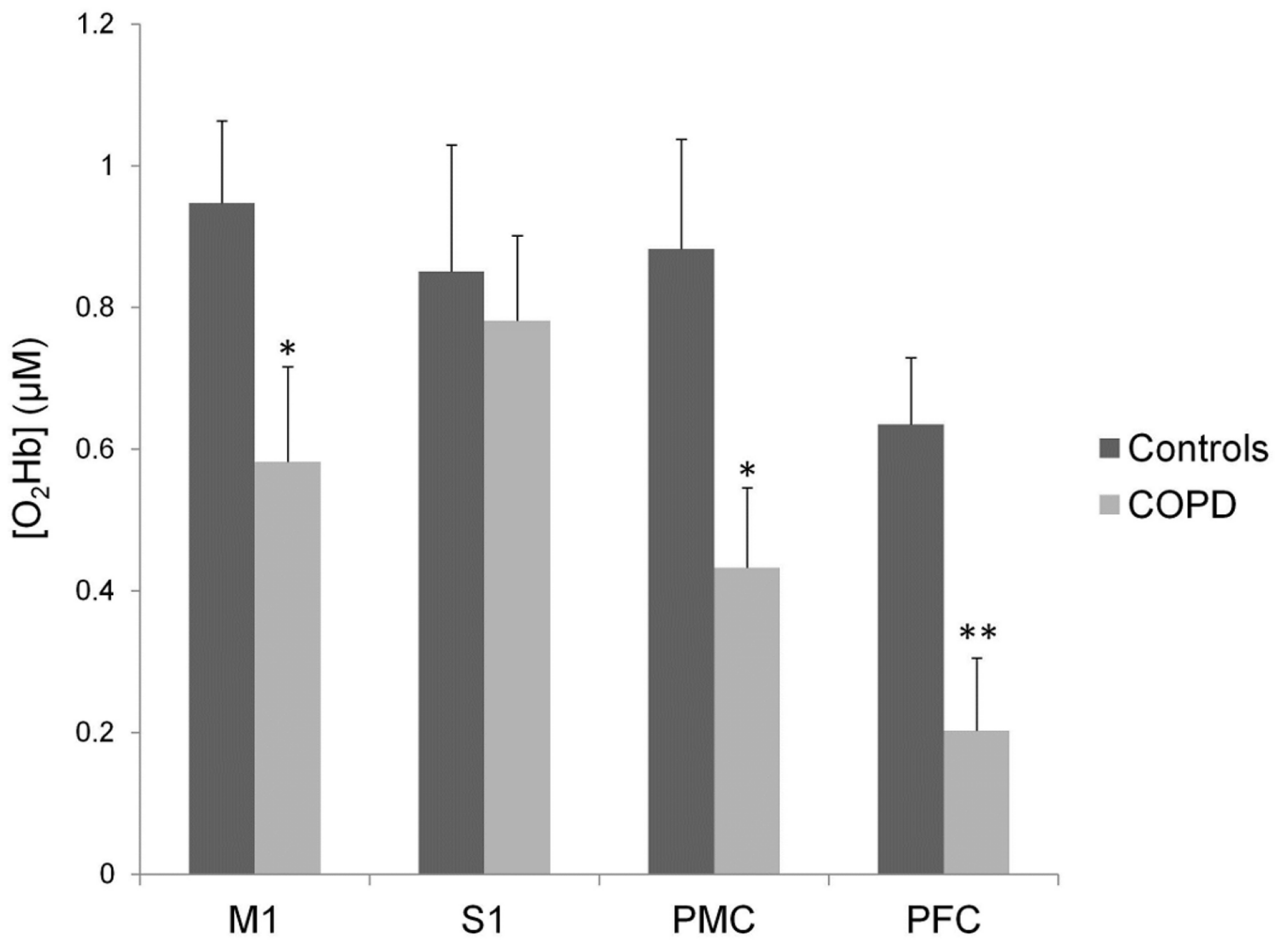
HbO changes during maximal voluntary contractions over primary motor (M1), primary sensory (S1), premotor (PMC) and prefrontal (PFC) cortex areas. * p<0.05 and ** p<0.01 significantly different from controls.

The regional HbO during MVCs is shown in Figure 3. Compared with controls, patients showed significantly lower HbO changes over M1 (t = 2.1, p<0.05), PMC (t = 2.3, p<0.05) and PFC (t = 3.1, p<0.01). In contrast, HbO changes during MVCs were comparable between patients and controls over S1 (t = 0.3, p = 0.74).

### Submaximal voluntary contractions (SVCs)

**Figure 4:**
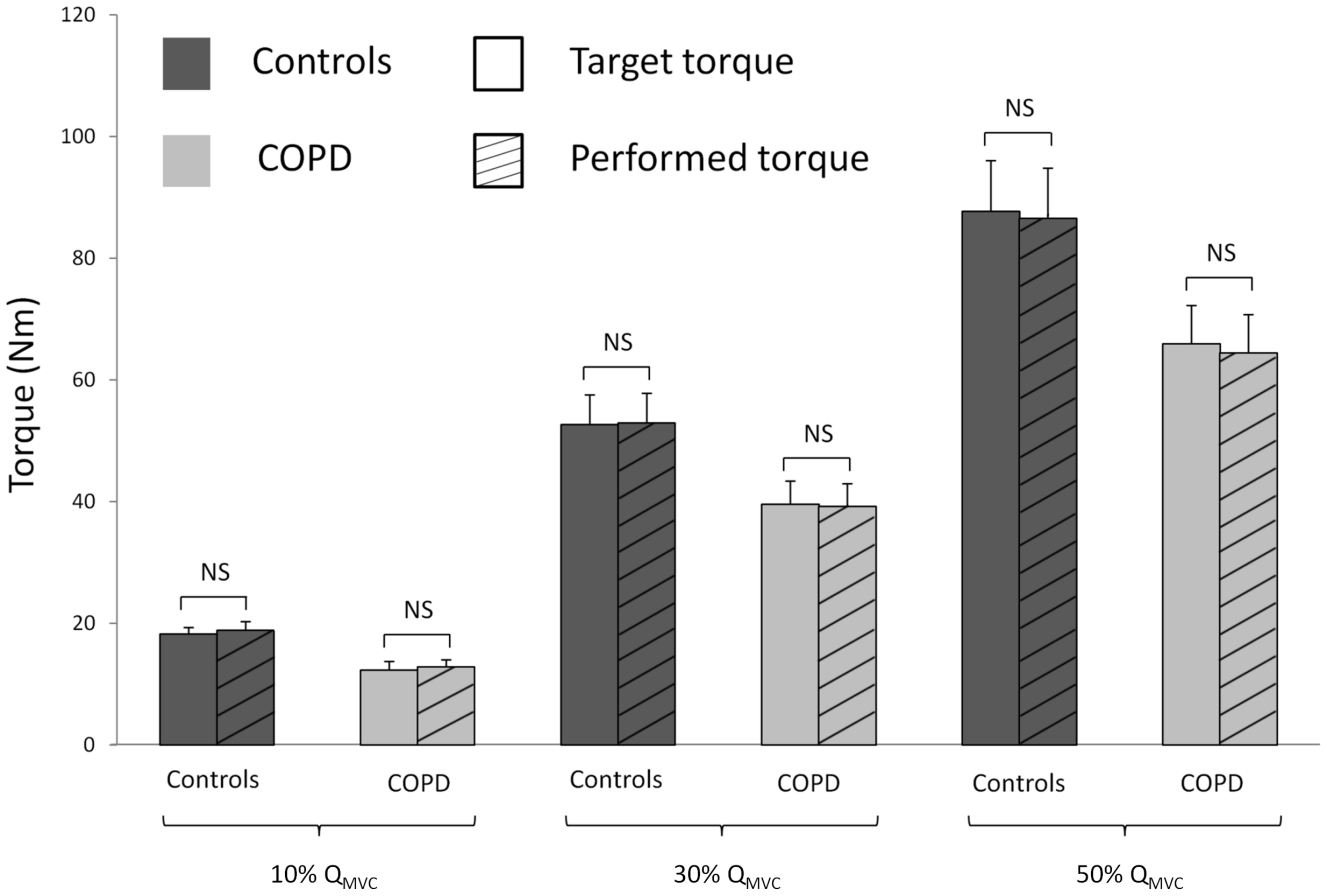
Performed torque versus target torque during submaximal voluntary contractions at 10, 30 and 50% of maximal quadriceps torque (Q_MVC_). NS: Non-significant difference between target and performed torque.

Task matching during SVCs was checked by comparing the performed torque with the target torque (Figure 4). No significant differences were found between performed and target torques for patients or controls at the three submaximal torque levels (F ranged from 0.31 to 2.22, p ranged from 0.15 to 0.74). In contrast, the Inaccuracy_index_ was significantly higher in patients compared with controls for all submaximal torque levels (F = 7.99, p<0.001). At 10, 30 and 50% of Q_MVC_, the Inaccuracy_index_ was 7.04±0.59 vs 5.15±0.62, 4.6±0.44 vs 3.46±0.58, and 4.83±0.47 vs 3.69±0.78 in patients and controls, respectively.

**Figure 5:**
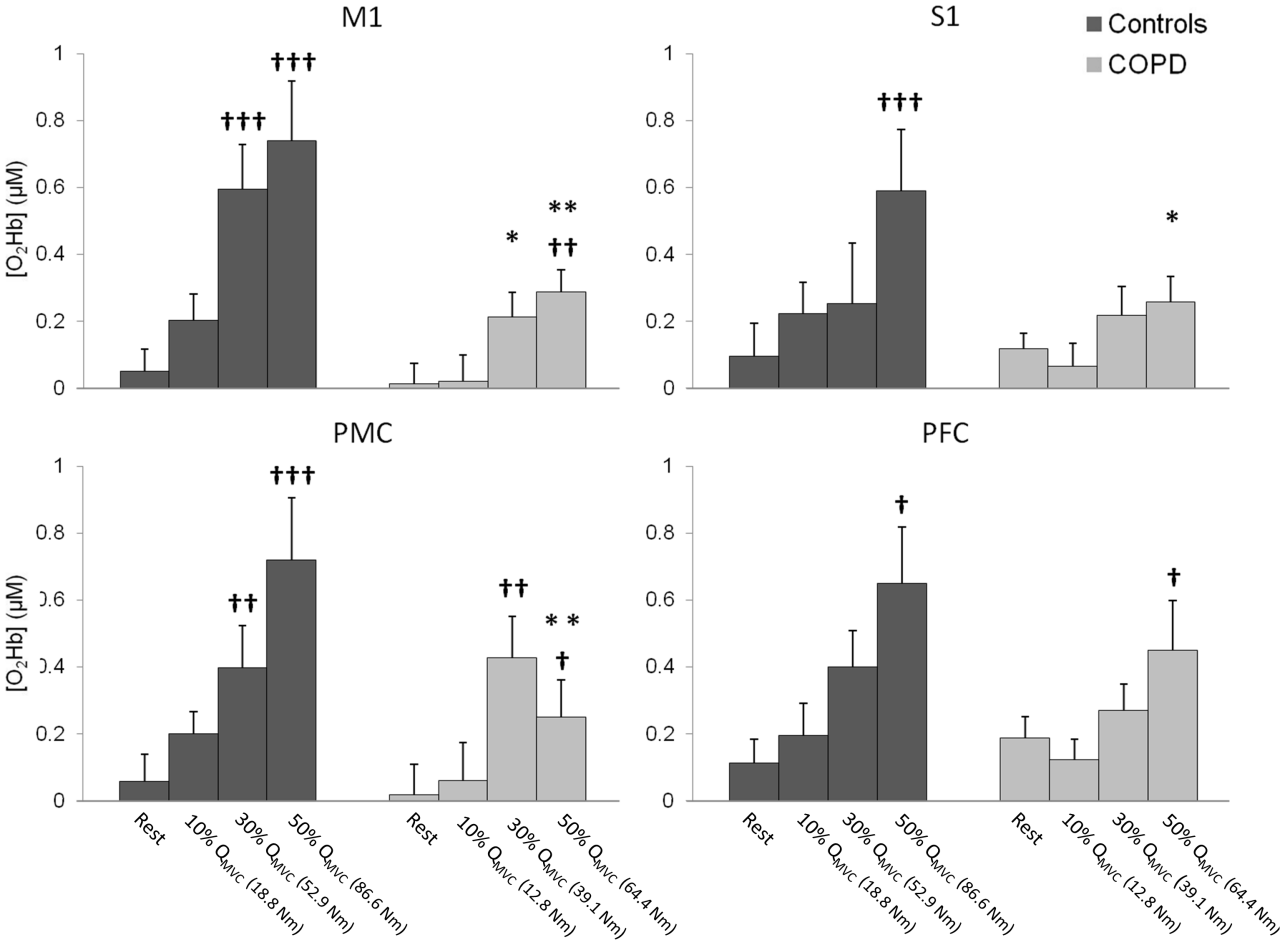
HbO changes during submaximal quadriceps contractions as a function of % of maximal quadriceps torque (Q_MVC_) over primary motor (M1), primary sensory (S1), premotor (PMC) and prefrontal (PFC) cortex areas. Values in parenthesis on the x axis indicate the mean torque performed at the given % of maximal quadriceps torque. Significant differences from rest: ^†^ p<0.05 ^††^ p<0.01 and ^†††^ p<0.001. Significant differences between controls and patients: * p<0.05, ** p<0.01 and *** p<0.001.

The regional HbO as a function of torque level is shown in Figure 5.

Over the M1 area, HbO was significantly increased compared with resting values, from 30% of Q_MVC_ in controls (p<0.001) and from 50% of Q_MVC_ in patients (p<0.01). Compared with controls, patients showed significantly lower HbO changes at 30% and 50% of Q_MVC_ (respectively, p<0.05 and p<0.01).

Over the S1 area, HbO was significantly increased compared with resting values, from 50% of Q_MVC_ in controls (p<0.001). In patients, HbO did not change significantly whatever the submaximal torque (p ranged from 0.34 to 0.49). In addition, at 50% of Q_MVC_, HbO changes were significantly lower in COPD patients than in controls (0.26±0.09 vs 0.59±0.18 µM, p<0.05).

Over the PMC area, HbO was significantly increased compared with resting values, from 30% of Q_MVC_ in patients and controls (systematically p<0.01). Compared with controls, patients showed lower HbO changes at 50% of Q_MVC_ (0.25±0.13 vs 0.72±0.12 µM, p<0.01).

Over the PFC area, HbO was significantly increased compared with resting values, at 50% of Q_MVC_ in patients and controls (systematically p<0.05). There was no difference in HbO changes between patients and controls for any submaximal torque level (F ranged from 0.75 to 0.9, p ranged from 0.35 to 0.53).

**Figure 6:**
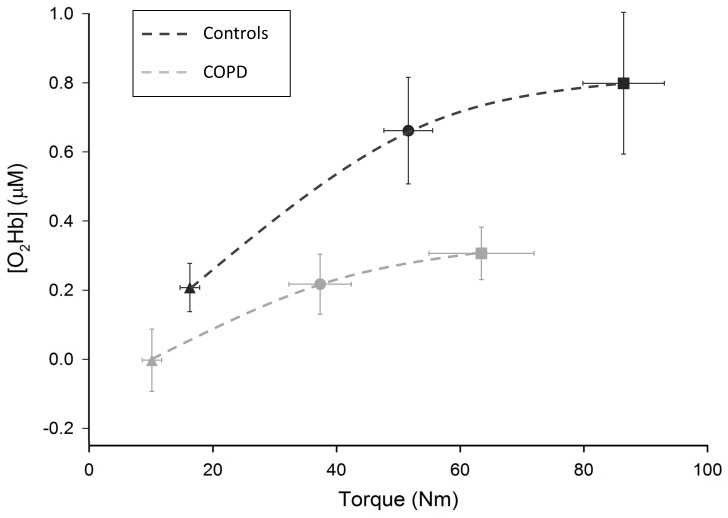
HbO changes over M1 as a function of absolute torque value at 10 (triangular shape), 30 (circular shape) and 50% (square shape) of the maximal voluntary torque.

The impact of the patients' lower absolute torque values compared with controls on HbO changes was checked using an ANCOVA. Consistently with respect to Figure 6 and adjusting for Q_MVC_, HbO remained significantly lower over M1 at 30% and 50% of Q_MVC_ in patients compared with controls (all p<0.05). Similarly, the observed effects in HbO changes over the S1, PMC and PFC areas were unaffected when Q_MVC_ was added as a covariable: HbO changes remained significantly lower over the S1 and PMC areas in patients at 50% of Q_MVC_ (all p<0.05), but comparable between the patients and controls over the PFC area (F ranged from 0.01 to 2, p ranged from 0.17 to 0.99).

## Discussion

The present study is the first to assess the neural correlates of quadriceps contractions in COPD patients. The main findings were lower HbO changes over the M1, PMC and PFC areas during maximal voluntary contractions in the COPD patients compared with controls. In addition, the COPD patients showed lower HbO changes than controls over the M1 area at 30% and 50% of Q_MVC_ and over the S1 and PMC areas at 50% of Q_MVC_. Last, the COPD patients exhibited greater torque fluctuations around the target than controls.

The COPD patients exhibited 24.8% lower muscle force than healthy controls. This is consistent with the usual torque deficit reported in the literature in moderate COPD patients, which ranges from 20% to 30% [37]. The neural correlates of quadriceps torque were simultaneously recorded with the non-invasive neuroimaging fNIRS technique [22], [25] over major cortical areas for movement generation. Our results show smaller HbO increases over the M1, PMC and PFC areas in the COPD patients during MVCs. These results cannot be due to oxygen desaturation because the exercise did not induce SpO_2_ changes. Similarly, it may not be explained by lower resting cerebral blood flow due to resting blood gases abnormalities because cerebrovascular reactivity to hypoxemia (increase in cerebral blood flow when PaO_2_ decreases) is preserved in COPD [38], [39]. According to the neurovascular coupling principle (as previously explained in the methods section), the data thus obtained with the fNIRS technique suggest a smaller local hyperoxygenation at the cortex in COPD patients compared with healthy controls. These results support lower neural activity in these patients, which would explain the decreased voluntary torque via reduced cortical motor output, and is coherent with the cerebrovascular damage and gray matter deficit described in the literature [8], [9].

During the submaximal voluntary contractions, we found a smaller HbO increase in the patients over the three main cortical areas of the frontal lobe involved in the execution and control of visual-motor tasks [40], at 30 and 50% of Q_MVC_ over the M1 area, and at 50% over the PMC and S1 areas. These results complete and support the findings of Vivodtzev et al. [17], who indirectly showed lower activation in COPD for comparable submaximal force levels with the twitch interpolation technique. In parallel to the altered neural activity, we found an increase in the inaccuracy index for submaximal torque levels in the COPD patients compared with controls, indicating greater torque fluctuations around the target in patients. Such torque fluctuations, known as dysmetria, are classic signs of lesions in the cerebellum [41], a subcortical area whose main function is the control and coordination of movement and whose output travels to motor and premotor cortex [42]. Interestingly, the dysmetria reported in the patients did not impact the task matching, as they were able to reach the required target (mean values). Hence, to summarize, the COPD patients were able to reach the desired target at submaximal intensities but with lower motor drive and high fluctuations, indicating less efficient motor control.

Given the difference in absolute torque value between the COPD patients and controls, we sought to ensure that the lower neural activity did not result from the lower muscle torque developed by the patients. As shown in Figure 6, for any given absolute torque value, increases in HbO were always about twice lower in the patients over M1. This agrees with the analysis of covariance, which indicated that adjusting for maximal voluntary torque had no impact on the difference in HbO changes between the COPD patients and controls. Taken together, these results provide new insight into the functional limitations in COPD patients, as the lower neural activity (lower increase in HbO) cannot be explained by either lower muscle torques or a lack of patient motivation or cooperation.

"In a previous study, Higashimoto et al. [24] recorded neural activity over the PFC area during a whole-body exercise that induced an increase in dyspnea score in both COPD patients and controls during testing, with the increase being higher in COPD. The authors reported a clear tendency toward smaller HbO changes in the COPD patients compared with healthy controls, although it did not reach the significance threshold. In addition, they reported correlations between the increase in dyspnea score and the increase in PFC activity during the exercise testing. These results raised the possibility of lower neural activity during whole-body exercise in COPD that might have been hidden by the greater increase in dyspnea-induced PFC activation [24]. Our findings are consistent with and complete the results of Higashimoto et al. [24], because a local exercise carried out without any dyspnea confirmed that the COPD patients had lower cortical activity."Several factors have been suggested to explain the cerebral alterations in COPD but the exact mechanisms remain unclear. These factors notably include inflammation, oxidative stress, hypoxemia and vascular disease [43]. In accordance with other studies [10], we report cerebral alterations in stable non-hypoxemic COPD patients, ruling out a determining role for hypoxemia. Understanding the mechanisms of the brain impairment in COPD patients has become a major issue. Our results provide new insight into the extrapulmonary effects of COPD on the brain and suggest new directions for research in order to optimize treatment for muscle force recovery in COPD. Further, they suggest the interest of early physical activity for COPD patients, given the potential effects of exercise on cerebral plasticity and neuroprotection [44], although this has yet to be specifically investigated in COPD.

In summary, COPD patients showed lower HbO changes over cortical motor areas during maximal and submaximal voluntary contractions of the knee extensors. This impairment was associated with a decrease in the maximal voluntary torque and altered motor control. The results provide the first evidence that the knee extensors of patients with stable moderate COPD cannot be optimally driven by the brain. Our findings highlight a lower motor cortex activity during quadriceps contraction in COPD and are consistent with an involvement of the central nervous system in the COPD quadriceps torque impairment. To optimize muscle force recovery in COPD patients, interventions targeting neuroprotection and neuroplasticity must be strongly considered.
